# Pediatric robotic assisted laparoscopic nephropexy: case study

**DOI:** 10.1186/2193-1801-2-321

**Published:** 2013-07-17

**Authors:** Danesh Bansal, W Robert Defoor, Paul H Noh

**Affiliations:** Division of Pediatric Urology, Cincinnati Children’s Hospital Medical Center, 3333 Burnet Avenue, ML 5037, Cincinnati, OH 45229 USA

**Keywords:** Nephropexy, Nephroptosis, Partial nephroureterectomy, Pediatrics, Robotic assisted laparoscopic

## Abstract

We describe pediatric robotic assisted laparoscopic left nephropexy in a 12-year-old female for symptomatic nephroptosis after partial nephroureterectomy.

## Introduction

Nephroptosis has been defined as renal descent of 5-cm or more when a patient moves from a supine to an erect position, causing pain in the abdomen or flank Wein et al. (2011). Most commonly, nephroptosis is seen in thin women, affects the right side, and symptomatic in only 10-20% of cases Fornara et al. (1997); Hoenig et al. (1999); Hubner et al. (1994); El-Moula et al. (2008). Laparoscopic nephropexy was first introduced by Urban and colleagues in 1993 Urban et al. (1993). In 2001, Plas et al. reported their long-term follow-up for laparoscopic nephropexy to treat symptomatic nephroptosis Plas et al. (2001). Only 2 cases of robotic assisted nephropexy have been reported, both in adult female patients Boylu et al. (2009); Baldassarre et al. (2011). We report the first case of a pediatric robotic assisted laparoscopic left nephropexy, for symptomatic nephroptosis after partial nephroureterectomy for upper urinary tract duplication anomaly.

### Case report

**Figure 1:**
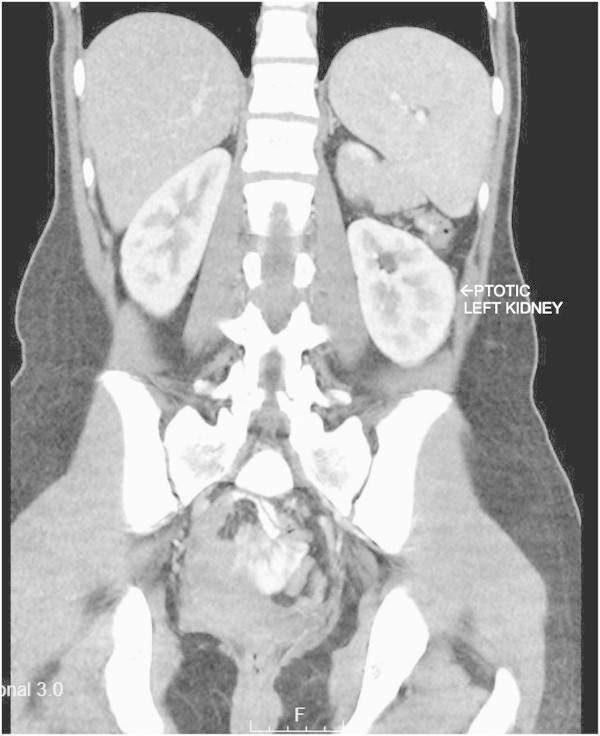
**Left ptotic kidney.**

**Figure 2:**
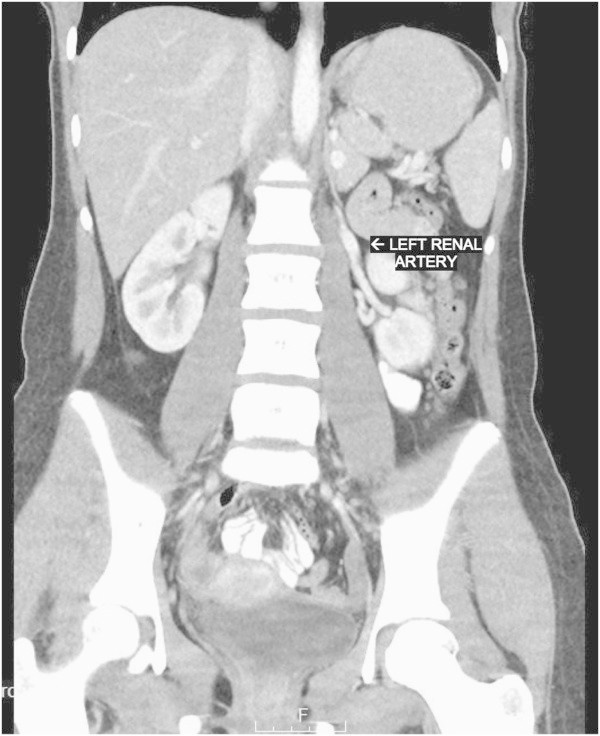
**Left renal artery.**

The patient is a 12-year-old female, who was born with a duplicated kidney and ectopic ureterocele. The patient underwent a left upper pole partial nephroureterectomy at 1 year of age. She subsequently underwent Deflux injection for persistent vesicoureteral reflux of the associated left lower pole renal moiety at 5 years of age. At 11 years of age, she developed debilitating left flank pain that caused her to discontinue all activities. The pain seemed to be positional and worse with physical activity. She underwent a rather extensive evaluation including blood tests for renal function and urinalysis that were all normal, a voiding cystourethrogram that showed no reflux, a renal ultrasound that showed no hydronephrosis, and a renal cortical scan that showed no acute inflammation but diminished relative differential function. Finally, a contrast computed tomography scan was performed to evaluate for a possible internal hernia. No hernia was identified, but the kidney was noted to be quite low in the retroperitoneum with the vessels oriented downward (Figures 1 and 2). She was followed conservatively, but the pain pattern did not improve. She was referred to the pain management clinic, and underwent physical therapy, but had no improvement. Her case was discussed in a multidisciplinary conference, and the diagnosis of nephroptosis was entertained. Robotic assisted laparoscopic nephropexy was thus offered to the family.

The patient was placed in a modified lateral decubitus position with the left flank upward. A transperitoneal approach was utilized. Open infraumbilical access was obtained through a curvilinear incision. One 8.5-mm robotic camera umbilical trocar, one 8-mm robotic instrument trocar in the midline below the xiphoid, one 8-mm robotic instrument trocar in the left lower quadrant, and one 5-mm assistant trocar in the lower midline were placed. The peritoneum lateral to the kidney was incised using a Maryland grasper and a hook electrode. The kidney was verified to be lower in the retroperitoneum, consistent with the CT scan, and not in a normal position after an upper pole partial nephrectomy in a duplication anomaly. Dense adhesions to the abdominal sidewall were divided. Once the kidney was fully mobilized, a decision was made to position the kidney near the splenic flexure. With a laparoscopic Kittner through the assistant port, the kidney was placed in the left upper quadrant. The fascia of the sidewall was sutured to the renal capsule with a series of 7 interrupted 3–0 prolene nephropexy sutures. Trocar site fascial incisions were closed with 4–0 Vicryl. There were no intraoperative complications or significant blood loss. The operative time was 243 minutes. A 10 French urethral catheter was left indwelling and removed on postoperative day 2. No other drains were used. The patient was discharged on postoperative day 4, after resolution of ileus.

At a 2-week postoperative visit, the patient was asymptomatic, and a renal ultrasound demonstrated the kidney to be much higher in the abdomen. At a 2-month postoperative visit, the patient reported resuming normal activities after postoperative activity restrictions were discontinued. The patient remains asymptomatic and without recurrence of pain after 18 months of follow-up.

## Discussion

To the best of our knowledge, we report the first case of a pediatric robotic assisted laparoscopic nephropexy, for symptomatic nephroptosis after open partial nephroureterectomy for upper urinary tract duplication anomaly. There have been 2 previous reports of a robotic assisted nephropexy in adult females, one of which was associated with a dismembered pyeloplasty Baldassarre et al. (2011); Boylu et al. (2009). Our patient was unique because she had a partial nephroureterectomy as an infant, when the kidney was noted to be orthotopic. The onset of a viral illness brought about flank pain near the surgical scar, subsequently leading to the finding of a ptotic left kidney. Symptomatic nephroptosis was diagnosed after all other etiologies were excluded.

The classical history for nephroptosis is flank or back pain that is typically aggravated after long periods of standing or walking Fornara et al. (1997). As seen in our patient, renography often shows a decrease in differential renal function O’Reilly & Pollard (1988). The underlying etiology is still unknown, but fixation of the kidney by fat, muscle, and connective tissue is typically decreased. Our case was atypical, due to fixation of the kidney in a ptotic position due to scar tissue after previous open kidney surgery. This highlights a rare clinical sequela of complete renal mobilization during surgery for upper urinary tract duplication anomalies. Once nephroptosis is symptomatic, treatment is warranted and in most cases conservative treatment relieves symptoms Hoenig et al. (1999). However, despite conservative treatment, some patients may remain symptomatic and require surgical intervention.

Laparoscopic nephropexy has been reported with less postoperative pain, less morbidity, and shorter hospitalization and convalescence when compared to the open approach Fornara et al. (1997); Hubner et al. (1994). Robotic nephropexy has been utilized for symptomatic nephroptosis due to more sophisticated instrument movement, magnified viewing, precise dissection and tissue handling, and comfortable positioning for the surgeon than conventional laparoscopy Boylu et al. (2009).

Robotic assisted laparoscopy has also become more common in the pediatric population as a minimally invasive alternative to classic open and laparoscopic approaches in an effort to minimize morbidity, while not comprising success. As the learning curve of the robotic assisted surgery is overcome in the pediatric population, a wider array of applications may be considered, including more challenging and complex procedures. Our patient was a felt to be good candidate for a robotic assisted laparoscopic procedure because of the benefits in suturing and due to a history of prior open retroperitoneal surgery, which may have required an extended recovery period if another open procedure was performed.

## Conclusion

Robotic assisted laparoscopic nephropexy can be safely and effectively performed in a pediatric patient. The technique may be added to the armamentarium of pediatric urologists performing minimally invasive surgery.

## Ethical approval

Written informed consent was obtained from the patient for publication of this Case Report/any accompanying images. A copy of the written consent is available for review by the Editor-in-Chief of this journal.
